# Cost-effectiveness of bedaquiline in MDR and XDR tuberculosis in Italy

**DOI:** 10.1080/20016689.2017.1283105

**Published:** 2017-02-17

**Authors:** Luigi R. Codecasa, Mondher Toumi, Anna D’Ausilio, Andrea Aiello, Francesco Damele, Roberta Termini, Alessia Uglietti, Robert Hettle, Giorgio Graziano, Saverio De Lorenzo

**Affiliations:** ^a^ Regional TB Reference Centre, Villa Marelli Institute/ASST Niguarda Ca’ Granda, Milano, Italy; ^b^ Faculty of Medicine, Public Health Department, Aix-Marseille University, Marseille, France; ^c^ Creativ-Ceutical, Milano, Italy; ^d^ Janssen-Cilag, Cologno Monzese, Italy; ^e^ Parexel International, London, UK; ^f^ Post-graduate Residency School in Hygiene and Preventive Medicine, University of Palermo, Palermo, Italy; ^g^ E. Morelli Hospital ASST, Reference Center for MDR-TB and HIV-TB, Sondalo, Italy

**Keywords:** Bedaquiline, cost-effectiveness, MDR tuberculosis, XDR tuberculosis, Italy

## Abstract

**Objective:** To evaluate the cost-effectiveness of bedaquiline plus background drug regimens (BR) for multidrug-resistant tuberculosis (MDR-TB) and extensively drug-resistant tuberculosis (XDR-TB) in Italy.

**Methods**: A Markov model was adapted to the Italian setting to estimate the incremental cost-effectiveness ratio (ICER) of bedaquiline plus BR (BBR) versus BR in the treatment of MDR-TB and XDR-TB over 10 years, from both the National Health Service (NHS) and societal perspective. Cost-effectiveness was evaluated in terms of life-years gained (LYG). Clinical data were sourced from trials; resource consumption for compared treatments was modelled according to advice from an expert clinicians panel. NHS tariffs for inpatient and outpatient resource consumption were retrieved from published Italian sources. Drug costs were provided by reference centres for disease treatment in Italy. A 3% annual discount was applied to both cost and effectiveness. Deterministic and probabilistic sensitivity analyses were conducted.

**Results**: Over 10 years, BBR vs. BR alone is cost-effective, with ICERs of €16,639/LYG and €4081/LYG for the NHS and society, respectively. The sensitivity analyses confirmed the robustness of the results from both considered perspectives.

**Conclusion**: In Italy, BBR vs. BR alone has proven to be cost-effective in the treatment of MDR-TB and XDR-TB under a range of scenarios.

## List of abbreviations

AIFA = Italian National Agency for Drugs

BR = background regimens

BBR = bedaquiline plus background regimen

DST = drug susceptibility testing

EMA = European Medicines Agency

HIV = human immunodeficiency virus

ICER = incremental cost-effectiveness ratio

Iv = intravenous

LBR = linezolid plus background regimen

LN = natural logarithm

LYG = life-year gained

MDR-TB = multidrug-resistant tuberculosis

miTT = modified intent-to-treat

NHS = Italian National Healthcare System

PSA = probabilistic sensitivity analysis

QALY = quality adjusted life year

SE = standard error

TB = tuberculosis.

XDR-TB = extensively drug-resistant tuberculosis

## Introduction

Multidrug-resistant tuberculosis (MDR-TB) is a disease caused by strains of mycobacterium tuberculosis resistant to treatment with isoniazid and rifampicin; extensively drug-resistant tuberculosis (XDR-TB) is caused by multidrug-resistant strains which are also resistant to treatment with fluoroquinolone and any of the injectable drugs used in second-line defence, such as amikacin, capreomycin, and kanamycin.[1–3] MDR-TB and XDR-TB pose serious threats to the progress made in the control of tuberculosis worldwide over the past decade.[1–3] In 2014, about 480,000 people, i.e. 5% of tuberculosis (TB) cases, in 105 countries, were diagnosed with MDR-TB, of which 190,000 died. A very high mortality rate was registered for XDR-TB worldwide; among 2685 XDR-TB patients in the 2012 cohorts of 41 countries (for whom outcomes were available), 809 (30%) of deaths were reported.[4,5]

The disease is not frequent in Italy, but it is a public health issue, considering that only 58% of patients with TB have a documented treatment success, while there is no such data available for MDR/XDR-TB. In 2014, the National Health Institute performed 2511 antibiograms of which 78 (3.1%) were MDR-TB cases and of them nine (11.5%) had XDR-TB.[6] These numbers may represent an underestimate due to increasing migration from regions where TB is endemic.[7]

MDR-TB and XDR-TB represent both a clinical and an economic burden worldwide. Combining direct and indirect costs, it has been estimated that in Europe, the average cost to treat drug-susceptible TB is €10,282 per patient. Costs increase to €57,213 for MDR-TB and to €170,744 for XDR-TB.[8] Particularly in Italy, the mean cost of a patient with TB is €9294 per year, while no detail is provided regarding the cost of MDR-TB and XDR-TB.[8]

Treatment of MDR-TB (Table 1) consists of an intensive phase of six to eight months with at least four active second-line drugs (including a quinolone and an injectable agent) added to any first-line drugs, to which the isolate is still susceptible, followed by a continuation phase, lasting at least 12 months after culture conversion.[9] Second-line drugs are more toxic and less tolerated than first-line drugs, and therefore require stricter clinical and laboratory monitoring of patients. Treatment success decreases as a function of the number and type of available drugs.[10,11] As MDR-TB and XDR-TB cases pose multiple clinical and management problems, it is highly recommended that these patients are referred to specialised centres in this field.[9]

**Table 1.** First- and second-line anti TB drugs.

Group	Drugs		Reference
Fluoroquinolones	Levofloxacin, moxifloxacin, gatifloxacin*		WHO [9]
Second lane injectable agents	Streptomycin, kanamycin, amikacin, capreomycin		WHO [9]
Other core second lane agents	Ethionamid* or prothionamid*, cycloserine* or terizidone*, linezolid, clofazimine*		WHO [9]
Add-on agents	D1	Ethambutol, pirazynamide, high dose isoniazid	WHO [9]
	D2	Bedaquiline, delamanid	WHO [9]
	D3	Para-aminosalicylic acid (PAS)*, imipenem-cilastatin, amoxicillin-clavulanate, meropenem,thioacetazone*	WHO [9]

*Drugs imported from abroad as not marketed in Italy

In 2013, the European Medicines Agency (EMA) granted marketing authorisation to bedaquiline, the first novel treatment with a new mechanism of action licensed for use in MDR-TB for over 40 years.[12] In 2014, the Italian Drugs Agency (AIFA) approved and reimbursed bedaquiline in Italy.[13]

The aim of this study is to estimate the cost-effectiveness of adding bedaquiline (add-on therapy at 24 weeks) to a background regimen (BBR) of at least three proven anti-TB drugs compared with background regimen (BR) alone, in the treatment of MDR-TB in Italy, from both the Italian National Healthcare System (NHS) and the Society perspective.

## Methods

**Figure 1.**
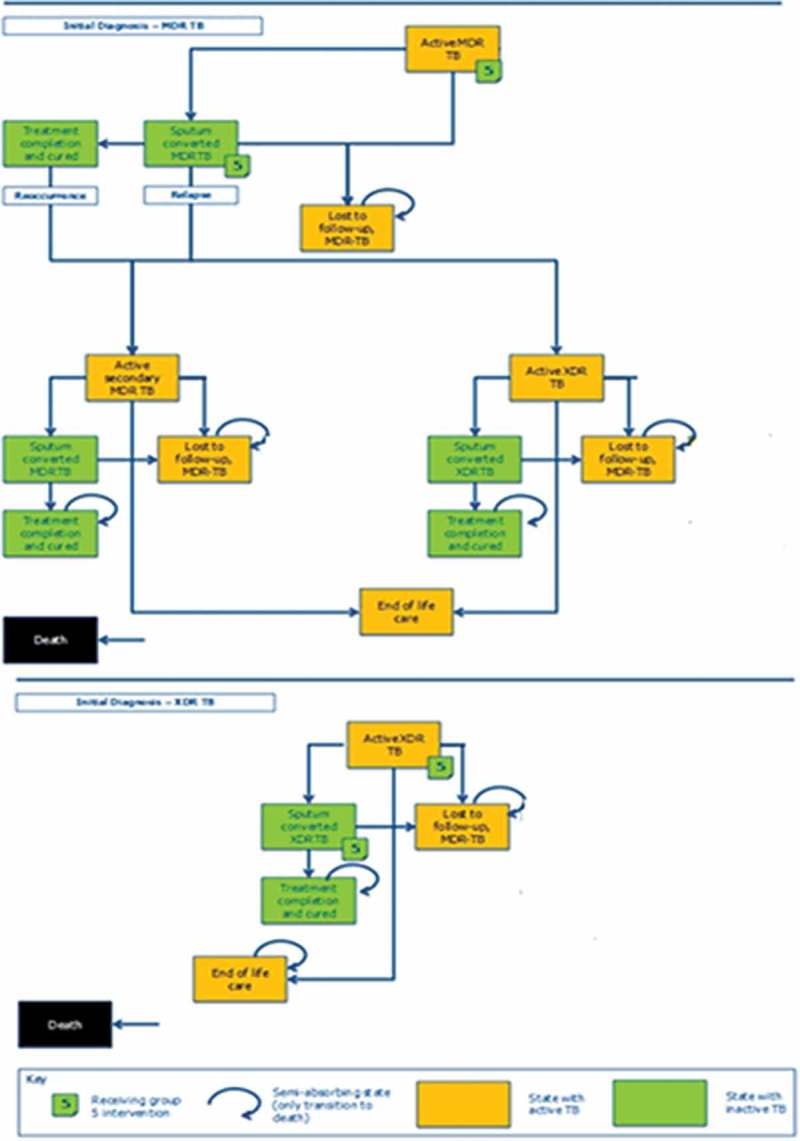
Markov model state structure. Note: modified from [14].

A previously developed cohort-based Markov model (Figure 1) [14] was adapted to the Italian Healthcare setting to evaluate the long-term economic and health benefits of achieving sputum culture conversion in patients with MDR-TB and XDR-TB.

The model structure is divided in two main branches: initial diagnosis of MDR-TB or XDR-TB. For patients with an initial diagnosis of MDR-TB, the model consists of eight core health states and six for XDR-TB patients. In the base case, treatment completion is achieved if patients do not fail treatment in the 12 months following sputum culture conversion, to World Health Organization (WHO) guidelines.[9] In the model, patients move between health states, over a fixed 28-day cycle period, in a 10-year timeframe, accruing cumulative costs, gaining life-year (LYG) and quality adjusted life-year (QALY). Each outcome is calculated midway through each cycle, based on the expected state occupancies, as the average of state occupancies at the start and end of a given cycle. The model considers the following time for treatment: for bedaquiline, an add-on therapy duration of 24 weeks; a period of six months (28 days per month) for intensive BR (intravenous); and of 20 months for culture post conversion, in each group of patients; for MDR-TB patients, a maximum period of 20 months is required for treatment (until the treatment is considered to have failed). Clinical trial data were sourced from published scientific literature (Table 2).[15–18]

### Assumptions

The following assumptions were considered in the model:

- The comparative efficacy of BBR versus BR in the treatment of patients with XDR-TB was based on data from the C208 study,[12,15] which enrolled patients with MDR-TB and pre-XDR-TB. Data on the use of bedaquiline in patients with XDR-TB were available through the C209 study,[12] but since this was an open-label, single-arm study, there was no information from which comparisons to placebo and BR could be made. It was therefore assumed that the relative benefits of treatment in the patient group with XDR-TB were equivalent to the relative benefits observed in C208.[12,15]
- The probability of experiencing all events, except for culture conversion and mortality in the general population, was assumed to be fixed over time.
- Relapses and recurrences were only permitted in patients with MDR-TB. Patients with XDR-TB were assumed to achieve conversion, complete treatment and return to general health or receive long-term palliative care for the remainder of the time horizon for those experiencing chronic TB.
- Withdrawal from treatment was considered only for patients lost to follow-up until death. No costs were applied to this patient group.
- Patients with secondary MDR-TB were assumed to experience a lower rate of culture conversion than patients with primary MDR-TB. The reduced rate of culture conversion was based on a comparison of the rate of conversion in patients with MDR-TB and pre-XDR-TB in the C209 study.[12]
- BR was used as an end-of-life care in patients failing treatment.

### Adaptation to the Italian setting

An expert panel of recognised Italian clinicians was interviewed through a structured questionnaire to gather information on: patients features (age, percentage of patients XDR-TB and MDR-TB); healthcare resources consumption (routine visits, instrumental tests, hospital admissions), according to treatment pathway; BR drugs used in Italy and consumption percentage for each drug in their hospitals^1^ ^1^Villa Marelli Institute / ASST Niguarda Ca’Granda, Milano, Italy.
^,^ ^2^ ^2^E. Morelli Hospital ASST, Sondalo, Italy. (Table A1) and the end-of-life care costs, which included the cost of BR drugs as well as the hospitalisation costs.

A 3% discount was applied to both costs and benefits.[19] Life-years mortality rates were adapted in the model using national statistics.[20]

In the analysis, the surgical interventions and related costs were not considered, because this is not the normal practice at this stage of the disease in Italy. Regarding hospitalisations, a mean time of 63 days for MDR-TB patients was considered, and 76 days for XDR-TB patients, for a weighted mean value of 66 days per patient (Table 3). The costs of healthcare resource consumption was estimated with national inpatient/outpatients tariffs and the threshold for ordinary hospitalization was used.[21,22]

**Table 2.** Treatment efficacy reported in clinical literature.

Study	Treatment arm	Sputum culture conversion at endpoint, *n* (%)	Hazard ratio (SE)	Sample size, *N*	Time point (weeks)	Population	Source
C208 study – stage II	BBR	52 (77.6%)	2.44 (0.57)	67	24	miTT	EMA,[12] Diacon [15]
	Placebo and BR	38 (57.6%)		66			

BR = background regimens; BBR = bedaquiline plus background regimen; miTT = modified intent-to-treat; SE = standard error

**Table 3.** Model adaptation to the Italian setting: outcomes.

Variables	Values	Reference
*Model setting*		
Time horizon (years)	10	Expert panel
Discount rate efficacy	3%	Fattore [19]
Discount rate cost	3%	Fattore [19]
*Patients features*		
Mean age (years)	25	Expert panel
Mean weight (kg)	70	Expert panel
Gender male	65%	Expert panel
Patients MDR-TB	79.3%	Expert panel
Patients XDR-TB	20.7%	Expert panel
*Hospital*		
Average number of days recovery for MDR-TB patients	63	Expert panel, data consumption
Average number of days recovery for XDR-TB patients	76	Expert panel, data consumption
Threshold limit for ordinary hospitalization	41	Ministero della Salute [21,22]

MDR-TB = multidrug resistant tuberculosis; XDR-TB = extensively drug-resistant tuberculosis.

For drugs (Table 4, Table A1), the maximum prices that the NHS reimburses, including 10% VAT, were considered. For BR drugs not marketed in Italy, the costs provided by the two reference centres^1^ ^,^ ^2^ were used; whereas, for bedaquiline, the ex-factory price (including 10% VAT) was used.[23]

**Table 4.** Model adaptation to the Italian setting: costs.

Variables	Values	Reference
*Daily drug cost*		
Bedaquiline (initial period)	€ 459.51	Farmadati [23]
Bedaquiline (regular)	€ 100.61	Farmadati [23]
Linezolid	€ 60.93	Expert panel
Mean background regimen	€ 37.76	Expert panel
*Outpatient*		
Outpatient medical consultation	€ 20.66	Ministero della Salute [21]
Complete blood count	€ 3.17	Ministero della Salute [21]
Sputum smear test	€ 0.52	Ministero della Salute [21]
Sputum culture test	€ 5.43	Ministero della Salute [21]
Chest X-ray	€ 15.49	Ministero della Salute [21]
Liver function tests	€ 5.62	Ministero della Salute [21]
Kidney function tests	€ 5.28	Ministero della Salute [21]
Audiometric test	€ 9.76	Ministero della Salute [21]
Cardiac monitoring	€ 11.62	Ministero della Salute [21]
Admission for MDR/XDR-TB with complications (DRG 79)	€ 5744.00	Ministero della Salute [21]
Admission for MDR/XDR-TB without complications (DRG 80)	€ 4422.00	Ministero della Salute [21]
Mean cost for ordinary hospitalization	€ 5330.00	Ministero della Salute [21,22]
Mean of extra bed cost	€ 158.00	Ministero della Salute [21,22]
*End-of-life costs*		
Monthly cost of palliative care (drugs and hospitalizations)	€5561.00	Expert panel; Ministero della Salute [21]
*Lost work productivity*		
Cost of productivity loss per day	€ 116.76	OECD [24]; Banca d’Italia [25] Working days calculator [26]
Employed patients	80.7%	ISTAT [27]
*Transmission*		
Mean number of contacts examined per case	6.5	Hardinge [28]; Ansari [29]
Cost of contact tracing per contact	€77.45	Expert panel; Ministero della Salute [21]
Average cost per exogenous case of TB	€9294.00	Diel [8]

DRG = disease related group tariff; MDR-TB = multidrug resistant tuberculosis; TB = tuberculosis; XDR-TB = extensively drug-resistant tuberculosis.

To estimate indirect costs, an average yearly gross salary of €25,804 was considered,[24,25] divided by the 221 working days estimated in 2014.[26] This resulted in an average daily cost of €116.76 due to productivity loss. The model considered 80.7% [27] employment rate, according to the national statistics of people aged 25–34 years in Italy (Table 3).

### Base case analysis

In the base case, BBR vs. placebo plus BR are compared. Outcomes are expressed in terms of incremental costs per LYG. The analysis was performed considering both the NHS and the societal perspective.

### Sensitivity analysis

Several sensitivity analyses, both univariate deterministic and probabilistic, were developed to test the strength of the results.

#### Deterministic sensitivity analyses

##### Utility values and QALYs

In the first analysis, QALYs were considered for the compared drugs. All patients were assumed to have active TB and experience the quality of life (QOL) of patients with active disease. To estimate QALYs, utility data from a previous study were used.[30,31]

##### Bedaquiline plus BR vs. linezolid plus BR

In the absence of randomised comparative studies for linezolid versus other options in the treatment of MDR-TB or XDR-TB, a recent systematic review of single-arm observational studies of linezolid in TB was used.[32] The model considered an eight-week duration for the add-on therapy with linezolid to BR regimens (LBR), as suggested by the expert panel. For linezolid, the price from the two Italian hospitals^1^ ^,^ ^2^ was used.

##### Cost of TB transmission

According to the Italian expert panel and literature data,[28,29] a mean number of 6.5 contacts examined per case were assumed. To estimate the cost of this tracking, the expert panel reported a mean of five chest-ray exams for an average cost of €77.45 per contact (Table 4). The average cost per exogenous case of TB was considered to be €9294.[8]

##### Price of bedaquiline ± 20%

The strength of the results for BBR vs. BR alone was evaluated, also according to a variable price of bedaquiline of ± 20%.

##### Hospitalisation over six months

In the last deterministic sensitivity analysis, the weight of the hospitalisation length on the cost-effectiveness results was assessed.

#### Probabilistic sensitivity analysis

A probabilistic sensitivity analysis (PSA) was developed to estimate the joint uncertainty of costs and effectiveness of key input parameters. A probabilistic distribution was assigned, reflecting both the central estimate (mean) of each parameter, its variance (standard error) and the anticipated shape of the data around its mean. The analysis was performed with 10,000 simulations. The results of the PSA are presented in cost-effectiveness acceptability curves.

## Results

### Base case analysis

Over the 10-year time horizon, for a mean patient assigned to BBR, the total discounted cost and LYGs were, respectively, €68,323 and 5.18, whereas, for BR, they were €51,615 and 4.17, respectively. The incremental cost-effectiveness ratio (ICER) was €16,639/LYG with the NHS perspective (Table 5).

**Table 5.** Base case cost-effectiveness analysis.

	Efficacy	Costs					
Variables	LYGs	Anti-TB drugs	Monitoring	Outpatient	Hospital	Indirect	Total
*NHS perspective*							
BBR	5.18	€ 48,807	€ 845	€ 339	€ 18,332	–	€ 68,323
BR alone	4.17	€ 28,632	€ 659	€ 358	€ 21,965	–	€ 51,615
ICER	€ 16,639 /LYG						
*Societal perspective*							
BBR	5.18	€ 48,807	€ 845	€ 339	€ 18,332	€ 21,650	€ 89,973
BR alone	4.17	€ 28,632	€ 659	€ 358	€ 21,965	€34,261	€ 85,875
ICER	€ 4081 /LYG						

BR = background regimens; BBR = bedaquiline plus background regimen; LYG = life year gained.

The analysis from the societal perspective shows that the incidence of indirect costs (productivity loss) is very high in both groups (€21,650 for the BBR group vs. €34,261 in the BR alone), and therefore the cost difference between the two groups of treatments is narrowed. The model calculated a mean cost per patient of €89,973 in BBR vs. €85,875 in BR alone. The ICER was €4081/LYG (Table 5).

Such results show that BBR is a cost-effective option in the treatment of MDR-TB and XDR-TB, both from the perspective of the NHS and the society.

### Sensitivity analysis

#### Deterministic sensitivity analysis

##### Utility values and QALYS

The model estimated a QALY gain of 4.36 for BBR vs. 3.29 for BR alone, with an ICER of €15,685/QALY from the NHS perspective and €3847/QALY from the society perspective. The results were very close to those of the base case analysis.

##### Bedaquiline plus BR vs. linezolid plus BR

With the NHS perspective, the model estimated a mean cost per patient treated with LBR of €54,423 and 4.48 LYGs, therefore, the ICER of BBR vs. LBR was €19,979/LYG from the NHS perspective. From a Societal point of view, the cost of LBR was €84,333, for an ICER of €8106/QALY.

##### Cost of TB transmission

The model showed that BBR is more favourable when the cost of transmission per patient infected is also taken into account. For each patient treated with BBR, there was 6.48% reduction of transmitted incremental case and 2.32% reduction of cases with an acquired resistant type. Considering the TB transmission, the mean additional costs per BBR patients of €3571 and of €4157 per BR patients were calculated. With this additional variable, the ICER becomes €16,055/LYGs for the NHS and €3497/LYGs for society.

##### Price of bedaquiline ±20%

With a 20% price increase per bedaquiline, results from the NHS perspective showed a BBR ICER of €21,244/LYG vs. BR alone. From the societal point of view, the ICER per BBR patient was €8685/LYG. With a 20% price decrease per bedaquiline, results from the NHS perspective showed an ICER of €12,034/LYG vs. BR alone, while from the societal point of view, results showed that BBR vs. BR alone was ‘dominant’ (more effective and less costly).

##### Hospitalization over six months

In the last deterministic sensitivity analysis, hospital costs were €28,224 per BBR patient and €36,999 per BR patient. Considering the NHS perspective, the total cost was €78,176 per BBR and €66,588 per BR alone, with an ICER of €11,541/LYG. From the societal perspective, the total cost was €99,826 per BBR and €100,848 per BR alone, so the results showed that BBR was the ‘dominant’ therapeutic pathway.

#### Probabilistic sensitivity analysis

**Figure 2.**
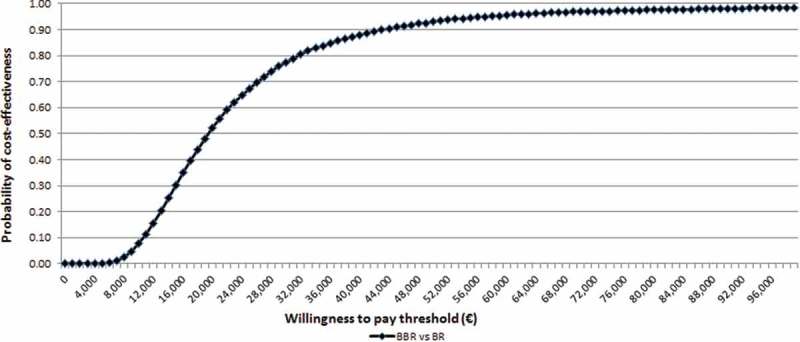
Cost-effectiveness acceptability curve for bedaquiline + BR versus BR: Italian NHS perspective. BR = background regimens; BBR = bedaquiline plus background regimen.

From the NHS perspective, the probability that BBR versus BR alone is cost-effective at an affordability threshold of €40,000 per LYG [19] and €60,000 [33] was 88% and 96%, respectively (Figure 2).

**Figure 3.**
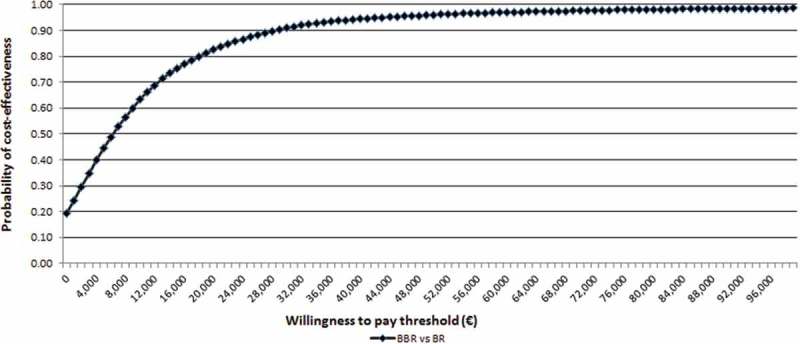
Cost-effectiveness acceptability curve for bedaquiline + BR versus BR: Italian societal perspective.

From the societal perspective, the probability that BBR is cost-effective at the above-mentioned thresholds was 94% and 97%, respectively (Figure 3). The strategy of BBR was cost-saving (and dominant) versus BR alone in 19% of probabilistic simulations. Considering a hospital stay of six months, the results of the PSA are more favourable, though closer to the base case scenario (with 63 days of hospital stay for MDR-TB patients and 76 for XDR-TB). Considering the societal perspective alone, the probability that BBR is dominant is 45%.

## Discussion and conclusion

BBR is highly likely to be cost-effective in most environments and its impact on costs will depend on price and the cost savings from retreatment. This saving effect will benefit countries, either with higher retreatment costs or higher current levels of treatment failures.[34,35] However, only very few incremental cost-effectiveness analyses on different MDR-TB treatment options have been published, and these refer to low or middle-income countries, where routine sputum culturing and drug susceptibility testing is not always available.[36]

For example, a recent review comparing MDR-TB interventions in Estonia, Peru, Philippines and Russia found that the cost per patient was US$10,880 (€8190), US$2423 (€1824), US$3613 (€2720) and US$14,657 (€11,033), respectively.[37] The best estimates of the cost per averted disability-adjusted life-year (DALY) were US$598 (€450), US$163 (€123), US$143 (€108) and US$745 (€561).[25,37] Cost varied according to different settings, for example, the lowest cost per DALY was found in Peru, where outpatient care, using a standardized regimen without drug testing, was principally employed.[37]

A preliminary ‘exploratory’ cost-effectiveness analysis on the practice of adding bedaquiline to the MDR-TB treatment regimen in six low- to middle-income countries showed that on a 20-month timeframe, BBR was considered cost-effective compared to a BR-alone strategy.[35]

Another study showed that in MDR-TB patients, BBR vs. BR alone in high income countries is a dominant strategy, with cost-savings of £11,434 (€14,184) and an additional 1.14 QALYs per patient.[16,25] The study focused on high cost savings due to fewer hospitalizations and less medical management in the outpatient setting.[16]

The results of our study demonstrate that, in Italy, adding bedaquiline to BR would be cost-effective through a range of different scenarios. In Italy, there are no official thresholds provided by the Ministry of Health or AIFA, but in general, thresholds between €25,000 and €60,000 per QALYs or LYGs are considered acceptable.[19,33]

Our analysis showed that ICERs were also under the lowest threshold and the results were confirmed in every sensitivity analysis; also when a 20% increase in the bedaquiline price was considered, it remained cost-effective (€26,481 for 188 tablets). The analysis was very conservative because it considered low hospital and outpatient costs, compared with other international and Italian analyses. Bocchino [38] showed a cost of TB case hospital management between €8509 in patients who die, up to €23,380 per patient transferred out. In [14], the cost of hospitalization in patients with BBR is twice the cost of the drugs and outpatient costs are similar to those of the drugs, whereas, in our analysis, we used very low (about nine times lower) hospital and outpatient costs.

Another important point is that the best ICERs are achieved with the societal perspective, because the disease has a big impact also on indirect costs and productivity loss in general. In the base case, cost savings of €12,844 per patient were obtained with BBR on indirect costs; these increased to €13,470 per patient when the cost of contact tracing and additional TB cases was considered. Such results are particularly relevant for infectious diseases like MDR-TB and XDR-TB. It is important to highlight that the analysis did not account for the out-of-pocket expenses (that is, non-reimbursed drugs and travel expenses to the hospital, as well as productivity loss by relatives/caregivers). On the other hand, it is important to highlight that bedaquiline is reimbursed and indicated for the specific treatment of MDR-TB and XDR-TB, while some drugs in the BR regimens are not always available in the Italian market. This forces hospitals to buy such drugs abroad with the risk of stock-outs.

As already stated,[14] the analysis has some limitations because the model was developed using assumptions that simplified the treatment pathway. At present, there are no data on the comparative efficacy of BBR versus BR alone in patients with XDR-TB. In the economic model, the relative efficacy of treatment from the C208 study (MDR-TB to pre-XDR TB) was used to evaluate the treatment effect in this patient group.[12,15] It is unclear whether the benefits observed in C208 can be extrapolated to a group with XDR-TB, although the results of the C209 open-label study, in which a substantial cohort of XDR-TB patients were treated with success, provide evidence that this extrapolation is justified.[12]

Any conclusions on the incremental cost-effectiveness of BBR vs. LBR should be treated with caution, due to the weaknesses in available data for either comparator.

Despite the limitations, our analysis shows that BBR vs. BR alone is a cost-effective strategy and its cost-effectiveness is even greater when considering the societal perspective.

## Supplementary Material

Appendix

## Appendix

**Table A1.** Percentage of use and daily cost for drugs used in Italy as Background Regimens.

Drug	Patients (%)	Dosage per day (mg) for a 70 kg patient	Daily costs of drugs for a 70 kg patient
**Group A**			
Levofloxacin (oral)	3.58%	1,000	€ 0.66
Moxifloxacin (oral)	78.42%	400	€ 1.10
**Group B**			
Amikacin (iv)	74.98%	750	€ 1.50
Capreomycin (iv)	1.60%	1,000	€ 44.30
**Group C**.			
Ethionamide	51.20%	750	€ 10.51
Prothionamide	41.34%	750	€ 10.51
Clofazimine (oral)	30.20%	100	€ 1.00
Terizidone (oral)	86.08%	750	€ 11.78
**Group D**			
Ethionamide (oral)	51.20%	750	€ 10.51
Protionamide (oral)	41.34%	750	€ 10.51
Para-aminosalicylic acid (oral)	42.56%	12,000	€ 10.41
Amoxicillin/clavulanate (oral)	42.14%	1750 / 250	€ 0.24
Meropenem (iv)	40.80%	3,000	€ 24.75
**Background Regimen average cost**			**€ 37.76**

Legend: iv = intravenous.

## References

[CIT0001] Nathanson E, Nunn P, Uplekar M (2010). MDR tuberculosis–critical steps for prevention and control. N Engl J Med.

[CIT0002] Raviglione MC, Smith IM. (2007). XDR tuberculosis–implications for global public health. N Engl J Med.

[CIT0003] World Health Organization (2010). Multidrug and extensively drug resistant TB (M/XD-TB). Global report on surveillance and response. Geneva, Switzerland: WHO Press, World Health Organization.

[CIT0004] World Health Organization (2013). Global tuberculosis report. Geneva, Switzerland: WHO Press, World Health Organization. WHO/HTM/TB/2013.11.

[CIT0005] World Health Organization (2015). Multidrug-resistant tuberculosis (MDR-TB). http://www.who.int/tb/challenges/mdr/mdr_tb_factsheet.pdf.

[CIT0006] European Centre for Disease Prevention and Control/World Health Organization Regional Office for Europe (2016). Tuberculosis surveillance and monitoring in Europe.

[CIT0007] Fattorini L, Mustazzolu A, Borroni E (2016). Italian multicentre study on resistance to antituberculosis drugs G: tuberculosis in migrants from 106 countries in Italy, 2008–2014. Eur Respir J.

[CIT0008] Diel R, Vandeputte J, de Vries G (2014). Costs of tuberculosis disease in the European Union: a systematic analysis and cost calculation. Eur Respir J.

[CIT0009] World Health Organization (2016). WHO treatment guidelines for drug-resistant tuberculosis. WHO/HTM/TB/2016.04.

[CIT0010] Migliori GB, Lange C, Centis R (2008). Resistance to second-line injectables and treatment outcomes in multidrug-resistant and extensively drug-resistant tuberculosis cases. Eur Respir J.

[CIT0011] Migliori GB, Lange C, Girardi E (2008). Fluoroquinolones: are they essential to treat multidrug-resistant tuberculosis?. Eur Respir J.

[CIT0012] European Medicines Agency (2013). Public assessment report: sirturo bedaquiline. EMA/CHMP/329898/2013.

[CIT0013] Agenzia Italiana del Farmaco Riclassificazione del medicinale per uso umano “Sirturo” (bedaquilina). Determina n.928, 2014. Gazzetta Ufficiale Serie Generale n.215 del 16-9-2014.

[CIT0014] Wolfson LJ, Walker A, Hettle R (2015). Cost-effectiveness of adding bedaquiline to drug regimens for the treatment of multidrug-resistant tuberculosis in the UK. PLoS One.

[CIT0015] Diacon AH, Lounis N, Dannemann B (2014). Multidrug-resistant tuberculosis and bedaquiline. N Engl J Med.

[CIT0016] Franke MF, Appleton SC, Bayona J (2008). Risk factors and mortality associated with default from multidrug-resistant tuberculosis treatment. Clin Infect Dis.

[CIT0017] Lee J, Lim HJ, Cho YJ (2011). Recurrence after successful treatment among patients with multidrug-resistant tuberculosis. Int J Tuberc Lung Dis.

[CIT0018] Tiemersma EW, van der Werf MJ, Borgdorff MW (2011). Natural history of tuberculosis: duration and fatality of untreated pulmonary tuberculosis in HIV negative patients: a systematic review. PLoS One.

[CIT0019] Fattore G (2009). Proposta di linee guida per la valutazione economica degli interventi sanitari. Pharmacoecon Ital Res Art.

[CIT0020] Istituto Nazionale di Statistica (2014). Mortality data of resident population.

[CIT0021] Ministero della Salute Remunerazione prestazioni di assistenza ospedaliera per acuti, assistenza ospedaliera di riabilitazione e di lungodegenza post acuzie e di assistenza specialistica ambulatoriale. Decreto 18/10/2012.; GU Serie Generale n.23 del 28-1-2013 - Suppl. Ordinario n. 8.

[CIT0022] Ministero della Salute (2008). La tubercolosi in Italia.

[CIT0023] Farmadati (2015). Compendio Farmaceutico Telematico.

[CIT0024] Organization for Economic Cooperation and Development (2014). Annual Average Wage.

[CIT0025] Banca d’Italia (2015). Exchange rates archives daily publication and historical series.

[CIT0026] (2014). Working days calculator in Italy.

[CIT0027] ISTAT (2014). Unemployment rate of resident population.

[CIT0028] Hardinge FM, Black M, Chamberlain P (1999). TB contact tracing in South Buckinghamshire from 1994-mid to 1998. Am J Respir Crit Care Med.

[CIT0029] Ansari S, Thomas S, Campbell IA (1998). Refined tuberculosis contact tracing in a low incidence area. Respir Med.

[CIT0030] Jit M, Stagg HR, Aldridge RW (2011). Treat Evaluation Team: dedicated outreach service for hard to reach patients with tuberculosis in London: observational study and economic evaluation. BMJ.

[CIT0031] Scalone L, Cortesi PA, Ciampichini R (2013). Italian population-based values of EQ-5D health states. Value Health.

[CIT0032] Cox H, Ford N (2012). Linezolid for the treatment of complicated drug-resistant tuberculosis: a systematic review and meta-analysis. Int J Tuberc Lung Dis.

[CIT0033] Messori A, Santarlasci B, Trippoli S (2003). Controvalore economico del farmaco e beneficio clinico: stato dell’arte della metodologia e applicazione di un algoritmo farmacoeconomico. Pharmacoecon Ital Res Art.

[CIT0034] Gunther G, Gomez GB, Lange C (2015). Availability, price and affordability of anti-tuberculosis drugs in Europe: a TBNET survey. Eur Respir J.

[CIT0035] Vassal A (2013). Cost-effectiveness of introducing bedaquiline in MDR-TB regimens—an exploratory analysis.

[CIT0036] Diel R, Hittel N, Schaberg T (2015). Cost effectiveness of treating multi-drug resistant tuberculosis by adding Deltyba to background regimens in Germany. Respir Med.

[CIT0037] Fitzpatrick C, Floyd K (2012). A systematic review of the cost and cost effectiveness of treatment for multidrug-resistant tuberculosis. Pharmacoeconomics.

[CIT0038] Bocchino M, Greco S, Rosati Y (2006). Cost determinants of tuberculosis management in a low-prevalence country. Int J Tuberc Lung Dis.

