# Supplementary material for: Cost-effectiveness of bedaquiline in MDR and XDR tuberculosis in Italy
Source: J Mark Access Health Policy. 2017 Feb 17;5(1):1283105. doi: 10.1080/20016689.2017.1283105 (PMC5328329; doi:10.1080/20016689.2017.1283105)
Supplement: Appendix [file zjma_a_1283105_sm9887.docx]

**Appendix**

Table A 1. Percentage of use and daily cost for drugs used in Italy as Background Regimens

| **Drug** | **Patients (%)** | **Dosage per day (mg) for a 70 kg patient** | **Daily costs of drugs for a 70 kg patient** |
| --- | --- | --- | --- |
| **Group A** | | | |
| Levofloxacin (oral) | 3.58% | 1,000 | € 0.66 |
| Moxifloxacin (oral) | 78.42% | 400 | € 1.10 |
| **Group B** | | | |
| Amikacin (iv) | 74.98% | 750 | € 1.50 |
| Capreomycin (iv) | 1.60% | 1,000 | € 44.30 |
| **Group C**. | | | |
| Ethionamide | 51.20% | 750 | € 10.51 |
| Prothionamide | 41.34% | 750 | € 10.51 |
| Clofazimine (oral) | 30.20% | 100 | € 1.00 |
| Terizidone (oral) | 86.08% | 750 | € 11.78 |
| **Group D** | | | |
| Ethionamide (oral) | 51.20% | 750 | € 10.51 |
| Protionamide (oral) | 41.34% | 750 | € 10.51 |
| Para-aminosalicylic acid (oral) | 42.56% | 12,000 | € 10.41 |
| Amoxicillin/clavulanate (oral) | 42.14% | 1750 / 250 | € 0.24 |
| Meropenem (iv) | 40.80% | 3,000 | € 24.75 |
| **Background Regimen average cost** | | | **€ 37.76** |

Legend: iv = intravenous
